# Runt‐related transcription factor 1 (Runx1) aggravates pathological cardiac hypertrophy by promoting p53 expression

**DOI:** 10.1111/jcmm.16704

**Published:** 2021-06-30

**Authors:** Dianhong Zhang, Cui Liang, Pengcheng Li, Lulu Yang, Zhengyang Hao, Lingyao Kong, Xiaoxu Tian, Chenran Guo, Jianzeng Dong, Yanzhou Zhang, Binbin Du

**Affiliations:** ^1^ Department of Cardiology The First Affiliated Hospital of Zhengzhou University Zhengzhou China; ^2^ Department of Cardiology Beijing Anzhen Hospital Capital Medical University Beijing China

**Keywords:** cardiac hypertrophy, heart failure, p53, Runx1

## Abstract

Cardiac hypertrophy and the resultant heart failure are among the most common causes of morbidity and mortality worldwide; thus, identifying the key factor mediating pathological cardiac hypertrophy is critically important for developing the strategy to protect against heart failure. Runx1 (Runt‐related transcription factor 1) acts as an essential transcription factor that functions in a variety of cellular processes including differentiation, proliferation, tissue growth and DNA damage response. However, relatively little is known about the role of Runx1 in heart, especially cardiac hypertrophy and heart failure. In the present study, we investigated the role of Runx1 in experimentally pathological cardiac hypertrophy. The in vitro model was induced by Ang II exposure to cultured neonatal rat cardiomyocytes, and the in vivo pathological cardiac hypertrophy models were induced by chronic pressure overload in mice. Runx1 expression is increased in heart tissues from mice with pressure overload–induced cardiac hypertrophy and in neonatal rat cardiomyocytes in response to Ang II stimulation. Moreover, knockdown of cardiac Runx1 alleviates the pressure overload–induced cardiac hypertrophy. Mechanistically, Runx1 activates the p53 signalling by binding to the p53 gene and promotes its transcription. Rescue experiments indicate that Runx1 promotes cardiac hypertrophy in a p53‐dependent manner. Remarkably, we demonstrated that Ro5‐3335 (a Runx1 inhibitor) acts as a potential therapeutic drug for treating pathological cardiac hypertrophy. In summary, we conclude that Runx1 is a novel mediator and therapeutic target for pathological cardiac hypertrophy.

## 1 INTRODUCTION

Recent epidemiology data have shown that heart failure is a high and steadily growing public health problem and a leading cause of mortality worldwide.^1^, ^2^ Pathological cardiac hypertrophy in response to various extracellular stress stimuli is a high‐risk factor for heart failure.^3^ Pathological cardiac hypertrophy, characterized by increased thickness of the heart muscle, apoptosis and fibrosis, is often associated with diseases such as hypertension, valvular disease and ischaemic heart disease and progressively leads to heart failure.^3^, ^4^ Despite the large body of literature outlining mechanisms of cardiac hypertrophy, there is no definite therapy to cure it. Identifying the key factor mediating cardiac hypertrophy and preventing the development of hypertrophy is critically important for reducing cardiovascular events.

The Runx gene family, encoding DNA‐binding α subunits that partner core binding factor β to form heterodimeric transcription factors, is an important regulator of biological processes.^5^ There are three members of the Runx family, including Runx1, Runx2 and Runx3.^5^ In mammals, Runx1 has essential regulator functions in a variety of cellular processes including proliferation, differentiation, tissue growth and DNA damage response.^6^, ^7^, ^8^, ^9^ To date, most research has focused on the role of Runx1 in the blood and cancer research fields.^10^ Relatively little is known about the role of Runx1 in heart, especially cardiac hypertrophy and heart failure. Interestingly, Runx1 was found to be increased experimentally in animal models of diabetic cardiomyopathy, pressure overload and dilated cardiomyopathy.^11^, ^12^, ^13^ More recently, Runx1 was reported to preserve left ventricular systolic function through improved SR‐mediated calcium uptake after myocardial infarction.^14^ At the cellular and molecular level, pathological cardiac hypertrophy is the result of a complex series of pathological processes including cell differentiation, proliferation, DNA damage, apoptosis, fibrosis and inflammation.^4^, ^15^, ^16^ We therefore hypothesized that Runx1 might play a prominent role in progression of cardiac hypertrophy.

Findings from our study demonstrate that expression of Runx1 was increased in pathological cardiac hypertrophy. Runx1 gain‐ and loss‐of‐function studies performed in vitro revealed that Runx1 promotes cardiomyocyte hypertrophy. Moreover, in vivo studies confirmed that knockdown of Runx1 gene expression in cardiomyocytes alleviates pathological cardiac hypertrophy. Mechanistically, Runx1 binds to the p53 gene and promotes its transcription. Through mediating the expression of p53, Runx1 controls the progression of pathological cardiac hypertrophy. Finally, targeting Runx1 is able to effectively inhibit progression of pathological cardiac hypertrophy.

## 2 MATERIALS AND METHODS

### 2.1 Animal models and treatment

All animal experiments were performed in accordance with the National Institutes of Health Guide for the Care and Use of Laboratory Animals and received the approval of the Animal Care and Use Committees of the First Affiliated Hospital of Zhengzhou University.

The adult male C57BL/6 mice (8 to 10 weeks old) were subjected to induce cardiac hypertrophy. Mice were immobilized using the single mouse restrainer, and 1 × 10^12^ vector genome (vg) per mouse of the prepared AAV9‐shRunx1 or its control (AAV9‐shCtrl) was injected through the tail vein. Transverse aortic constriction (TAC) was performed after two weeks subsequent to the transduction of the virus as previously described.^17^ Briefly, mice were fixed in a supine position after anaesthetized with sodium pentobarbital (90 mg/Kg; Sigma‐Aldrich) via an intraperitoneal injection. The left side of the chest was opened to expose the thoracic aorta, and the transverse aorta was then constricted by ligating the aorta with a 7‐0 nylon suture around a 26/27‐gauge syringe needle placed parallel above the vessel. The needle was rapidly removed after the procedure, and the chest was subsequently closed in layers. Mice in the sham group underwent all operation procedures except for the ligation.

We also injected Ro5‐3335 for 3 weeks from 1 week after TAC surgery by implanting Osmotic Minipumps (ALZET, model 2004) into the abdomens of mice.^18^ Control mice underwent the same procedure, except that the respective pumps were filled only with vehicle.

### 2.2 Echocardiography

Mice were anaesthetized with 1.5% isoflurane, and echocardiography was conducted by a MyLab 30CV ultrasound system (Biosound Esaote, Inc) by using a 15‐MHz probe as described previously. Briefly, after the chests of mice were shaved, the left ventricular end‐diastolic diameter (LVEDd), ejection fraction (EF) and LV end‐systolic diameter (LVESd) were determined through M‐mode tracings derived from the short axis of the LV at the level of the papillary muscles. The fraction shortening (FS) was calculated using the following formula: FS (%) = (LVEDd‐LVESd)/LVEDd × 100%.

### 2.3 Histological analysis

Hearts were sectioned and stained with haematoxylin‐eosin staining (HE) to measure myocyte cross‐sectional areas (CSAs) or picro Sirius red (PSR) to detect the degree of cardiac interstitial fibrosis. Image analysis was performed using a digital image analysis system (Image‐Pro Plus, version 6.0).

### 2.4 Cell culture and treatment

Cultured neonatal rat cardiomyocytes (NRCMs) were isolated from 1 ~ 3‐day‐old Sprague Dawley (SD) rats as described previously.^19^ After culture with serum‐free DMEM/F12 for 12 hours, cardiomyocytes were stimulated with Ang II (1 μmol/L) for 48 hours to induce cardiomyocytes hypertrophic model. In some experiments, cells were pre‐infected with Ad Runx1 or Ad shRunx1 for 12 hours to mediate the target gene expression before stimulation with Ang II.

### 2.5 Immunofluorescence staining

Immunofluorescence staining was performed to determine the surface area of the NRCMs. Briefly, after stimulation, the NRCMs were subsequently fixed with 4% formaldehyde for 15 minutes, permeabilized with 0.1% Triton X‐100 in PBS for 5 minutes and stained with a‐actinin (Cell Signaling Technology) followed by a fluorescent secondary antibody. Image‐Pro Plus 6.0 software was used to measure the cell surface area.

### 2.6 Luciferase Assay

The luciferase reporter assay was performed as described previously.^20^ HEK293T cells were seeded in 24‐well plates. After 24 hours, the cells were transfected with PGL3‐Runx1‐luc with the indicated constructs. After 24 hours, the luciferase activity was measured using the Dual‐Luciferase Reporter Assay System (Promega, USA) according to the manufacturer's instructions.

### 2.7 Chromatin immunoprecipitation (ChIP) assay

ChIP assay has been described previously elsewhere.^20^ In brief, NRCMs infected with Ad Runx1 were cross‐linked with 1% formaldehyde for 10 minutes at temperature. Then, the cells were harvested in lysis buffer and dealt with pulse ultrasonication to shear DNA into fragment with 200‐500 bp fragment. After ultrasonication, the samples were divided equally and incubated with 10ug Runx1 antibody, 2ug non‐specific immunoglobulin G (IgG) antibody overnight at 4℃ with rotation. The next day, each sample was incubated with 40 μL protein A agarose beads (Thermo Fisher Scientific) for 3 hours at 4℃ with rotation. Then, the immunocomplexes were washed twice with lysis buffer. After the procedure, the precipitated DNAs were subjected to qPCR using primers shown in Table S1.

### 2.8 Western blotting

Protein was extracted from cardiac tissues or cell samples using RIPA lysis buffer. The cell lysate was fractionated by 10% SDS‐PAGE and subsequently transferred to PVDF membranes (Millipore) and incubated with different primary antibodies overnight. After incubated with the primary antibodies, membranes were washed with TBS‐T and incubated with the corresponding secondary antibodies for 1h at room temperature. Finally, the blots were scanned by a Bio‐Rad ChemiDoc XRS+system (Bio‐Rad). The following antibodies were used: Runx1 antibody (Cell Signaling Technology; #4334; dilution 1:1000), p53 antibody (Abcam; #ab26; dilution 1:1000), p‐p53 antibody (Abcam; #ab223868; dilution 1:2000), Noxa antibody (Cell Signaling Technology; #14766; dilution 1:1000), Bax antibody (Cell Signaling Technology; #5023; dilution 1:1000), Puma antibody (Cell Signaling Technology; #24633; dilution 1:1000), Anp antibody (Abcam; #ab181242; dilution 1:10 000) and Gapdh antibody (Abcam; #ab8245; dilution 1:50 000).

### 2.9 Quantitative real‐time PCR

Total mRNA was isolated from the ventricular tissues of cell samples with TRIzol reagent (Invitrogen), and the cDNA was reversely transcribed using the Transcriptor First Strand cDNA Synthesis Kit. The relative expression of indicated genes was detected with the SYBR Green PCR Master Mix by using quantitative real‐time PCR. The expression levels were normalized to GAPDH. The primers used in the experiments are shown in Table S1.

### 2.10 Statistical analysis

All experiments data were present as the mean ± SEM. Student's two‐tailed *t* test was used to compare the means of two‐group samples, and multiple group comparisons were performed by one‐way ANOVA using Tukey post hoc test. All statistical analyses were performed with SPSS 21.0 software. *P* < .05 was considered significant.

## 3 RESULTS

### 3.1 Runx1 expression is up‐regulated in pathological cardiac hypertrophy

**FIGURE 1:**
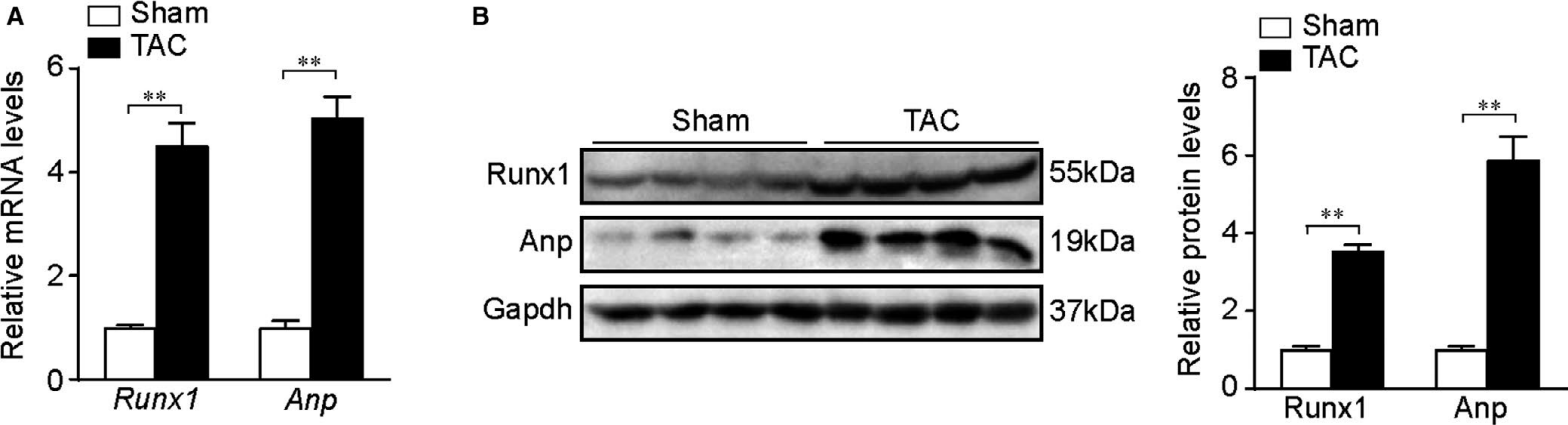
Runx1 expression in cardiac hypertrophy. (A) Representative mRNA levels of Runx1 and Anp (atrial natriuretic peptide) in heart tissues from sham or transverse aortic constriction (TAC) mice. n = 6 mice per group. (B) Representative immunoblot images and statistics of the protein levels of Runx1 and Anp (atrial natriuretic peptide) in heart tissues from sham or transverse aortic constriction (TAC) mice. n = 6 mice per group. *P < .05, **P < .01

We first explored Runx1 expression during cardiac hypertrophy in TAC‐induced cardiac hypertrophy mouse model. The mRNA and protein levels of ANP (atrial natriuretic peptide), the biomarker for cardiac hypertrophy, were remarkably increased in TAC mouse hearts (Figure 1A,B). Meanwhile, Runx1 mRNA and protein expression in hearts from TAC mice (4 weeks) were both markedly increased compared to the sham group (Figure 1A,B). Consistently, we stimulated NRCMs with 1uM Ang II for 48h and also checked Runx1 expression in vitro. Here, we found Runx1 mRNA and protein levels also significantly increased in NRCMs in response to hypertrophic stress in vitro (Figure S1A,B). These data indicate that Runx1 expression is up‐regulated in pathological cardiac hypertrophy.

### 3.2 Runx1 promotes Ang II–induced cardiomyocyte hypertrophy in vitro

**FIGURE 2:**
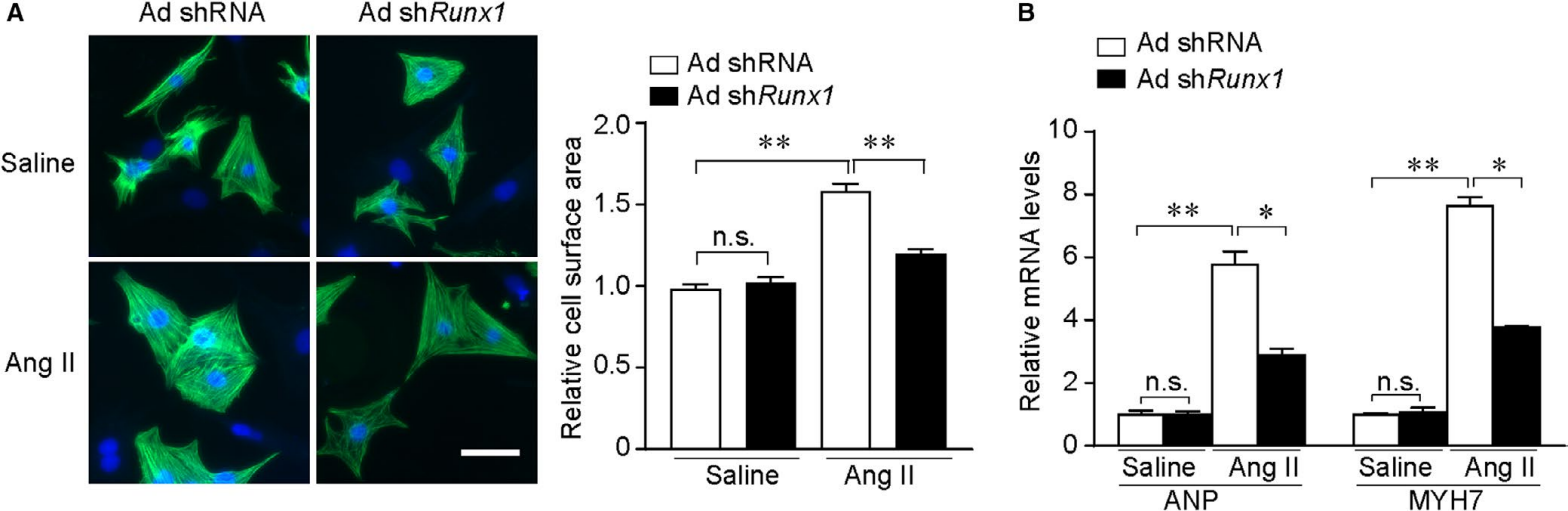
Knockdown of Runx1 alleviates cardiomyocyte hypertrophy induced by Ang II in NRCMs. (A) Left, representative images of NRCMs infected with the indicated adenoviruses and treated with saline or Ang II for 48 h. Cardiomyocytes were identified by a‐actinin staining (green), and nuclei were stained with DAPI (blue). (Scale bars, 50 μm). Right, quantitative results of the cell surface area of NRCMs (n ≥ 200 cells/group). (B) Representative mRNA levels of Anp and Myh7 in the indicated group. n = 4 samples per group. *P < .05, **P < .01

To characterize the pathological consequence of up‐regulated Runx1 expression in response to hypertrophic stimulation, we performed gain‐ and loss‐of‐function assays. Runx1 knockdown and overexpression were achieved by adenovirus Runx1 (Ad Runx1) and adenovirus shRunx1 (Ad shRunx1). The efficiency of adenovirus‐mediated gene expression was validated by Western blotting (Figure S2A). Runx1 knockdown in NRCMs ameliorated Ang II–induced cardiomyocyte hypertrophy, as indicated from decreased cell surface area (CSA) and mRNA levels of hypertrophic marker genes Anp and Myh7 (Figure 2A,B). Conversely, overexpression of Runx1 significantly promoted Ang II–induced cardiomyocyte hypertrophy and significantly increased expression of hypertrophic markers compared with the control group (Figure S2B,C). These in vitro results indicate that Runx1 positively regulated cardiomyocyte hypertrophy.

### 3.3 Runx1 knockdown attenuates TAC‐induced cardiac hypertrophy in mice

**FIGURE 3:**
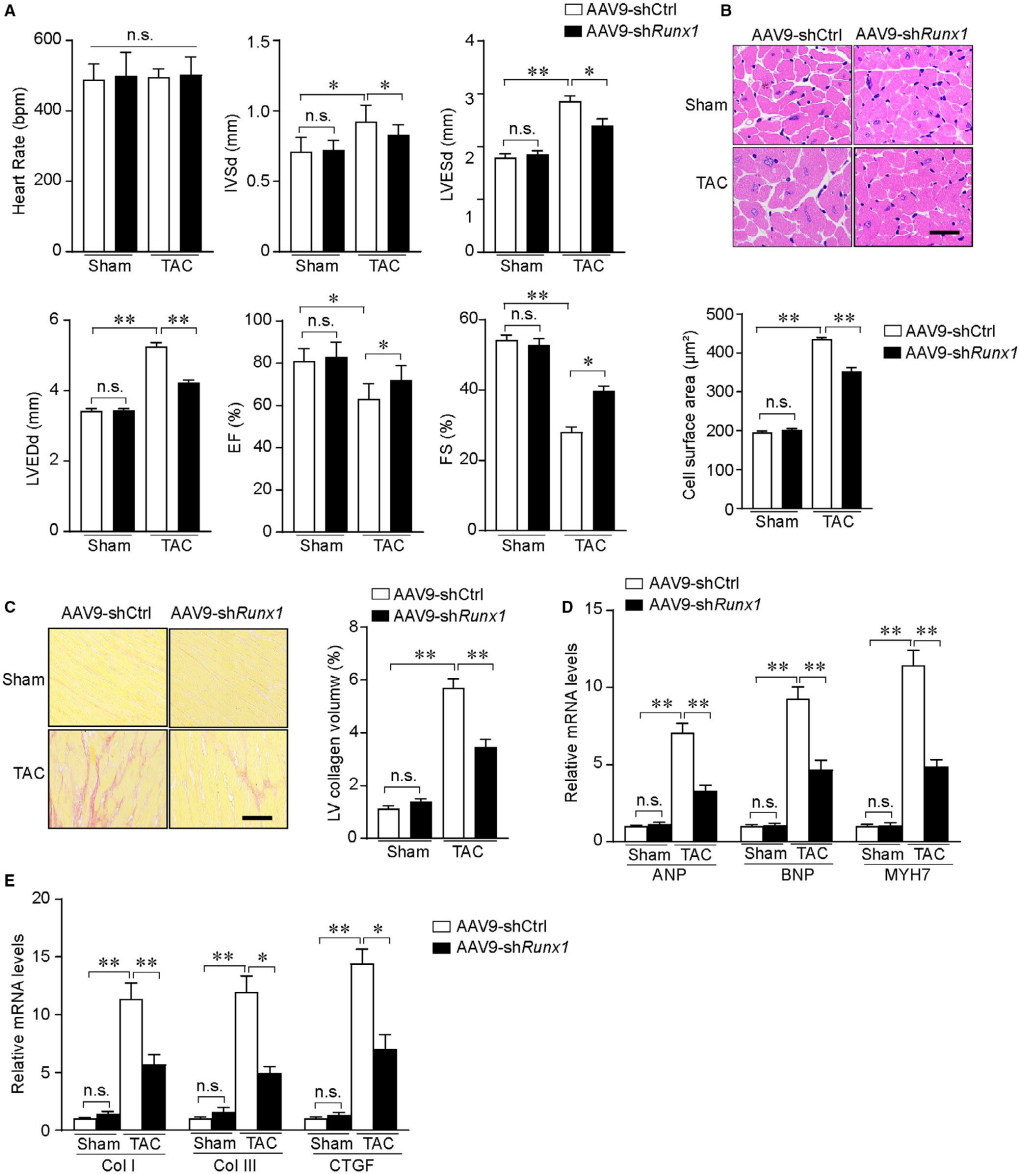
Cardiomyocyte‐specific Runx1 knockdown alleviates pressure overload–induced cardiac hypertrophy. (A) Statistical results of echocardiographic parameters in the indicated groups (n = 8‐10 mice per group). (B) Upper, representative images and of haematoxylin and eosin (HE)–stained heart sections from the indicated groups (scale bar, 50μm.), (n = 5‐7 mice per group). Low, statistical results of cardiomyocyte cross‐sectional area in the indicated groups, (n ≥ 50 fields per group). (C) Left, representative images of picro Sirius red (PSR)–stained heart sections from the indicated groups (scale bar, 100 μm.), (n = 5‐7 mice per group). Right, statistical results of left ventricle collagen volume determined by picro Sirius red staining. (D) The relative mRNA levels of atrial natriuretic peptide (Anp), B‐type natriuretic peptide (Bnp), and Myh7 in the left ventricle of mice from the indicated groups (n = 4 mice per group). (E) The relative mRNA levels of collagen I (Col I), collagen III (Col III) and connective tissue growth factor (CTGF) in the left ventricle of mice from the indicated groups (n = 4 mice per group). *P < .05, **P < .01

We used cardiac‐specific Runx1 knockdown (AAV9‐shRunx1) mice to characterize the role of Runx1 in TAC‐induced cardiac hypertrophy. The efficiency of AAV9‐mediated Runx1 expression in mice hearts was validated by Western blotting (Figure S3A). Echocardiography demonstrated that in wild‐type (WT) mice 4 weeks after TAC, the cardiac contractile function indexed by the left ventricular fractional shortening (FS) and ejection fraction% (EF%) were significantly decreased 4 weeks after TAC compared with the sham group (Figure 3A). Meanwhile, the interventricular septal thickness (IVS) at diastole, left ventricular systolic internal dimension (LVESd) and diastolic internal dimension (LVEDd) were increased 4 weeks after TAC (Figure 3A). When comparing between AAV9‐shRunx1 mice and AAV9‐shRNA group after TAC, we found Runx1 knockdown dramatically alleviated interventricular septal thickness, ventricular dilation and contractile dysfunction. Moreover, morphological examinations revealed that the degree of cardiomyocyte hypertrophy and interstitial fibrosis were alleviated in Runx1 knockdown mice as compared with controls in response to pressure overload (Figure 3B,C). Meanwhile, the mRNA levels of hypertrophic markers (ANP, BNP and MYH7) and fibrotic markers (collagen I, collagen III and Ctgf) were significantly decreased in the hearts of Runx1 knockdown mice compared with control mice in response to pressure overload (Figure 3D,E). Collectively, these data demonstrate that Runx1 contributes to the development of pathological cardiac hypertrophy in mice.

### 3.4 Runx1 promotes pathological cardiac hypertrophy through activation of p53

**FIGURE 4:**
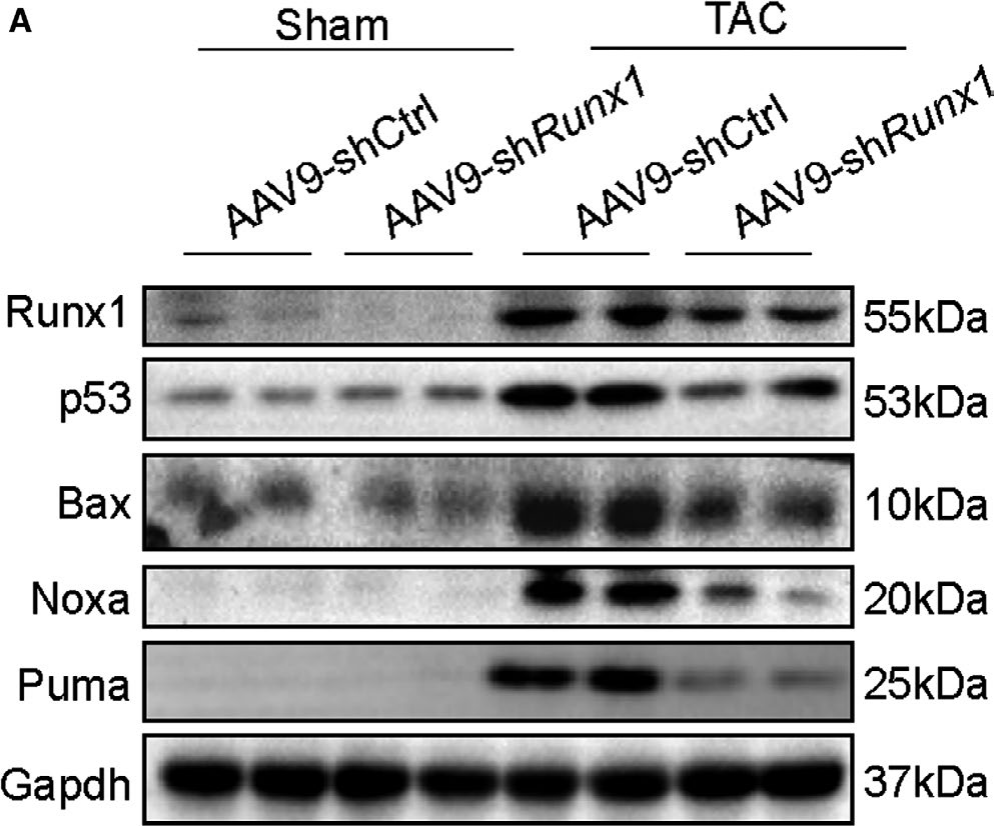
Runx1 contributes to cardiac hypertrophy by activating p53 signalling pathway. (A) Representative Western blots showing the protein levels of Runx1, p53, Noxa, Bax and Puma in mice heart tissues in the indicated groups, (n = 4 mice per group)

Since p53 has been identified as an Runx1‐inducible gene, and which has a crucial function in deteriorating cardiac hypertrophy, we focused on p53 as a candidate gene through which Runx1 might contribute to cardiac hypertrophy. We analysed the expression of p53 and the activation of p53‐related signalling pathways in mice hearts in response to hypertrophic stress. As demonstrated in Figure 4A, expressions of p53 and also its downstream target genes (Bax, Noxa, and Puma) were Runx1‐dependent. These data were consistent with our hypothesis that Runx1 promotes pathological cardiac hypertrophy by increasing p53 gene expression and subsequent downstream signalling pathways activation.

### 3.5 Runx1 transcriptionally regulates p53 expression

**FIGURE 5:**
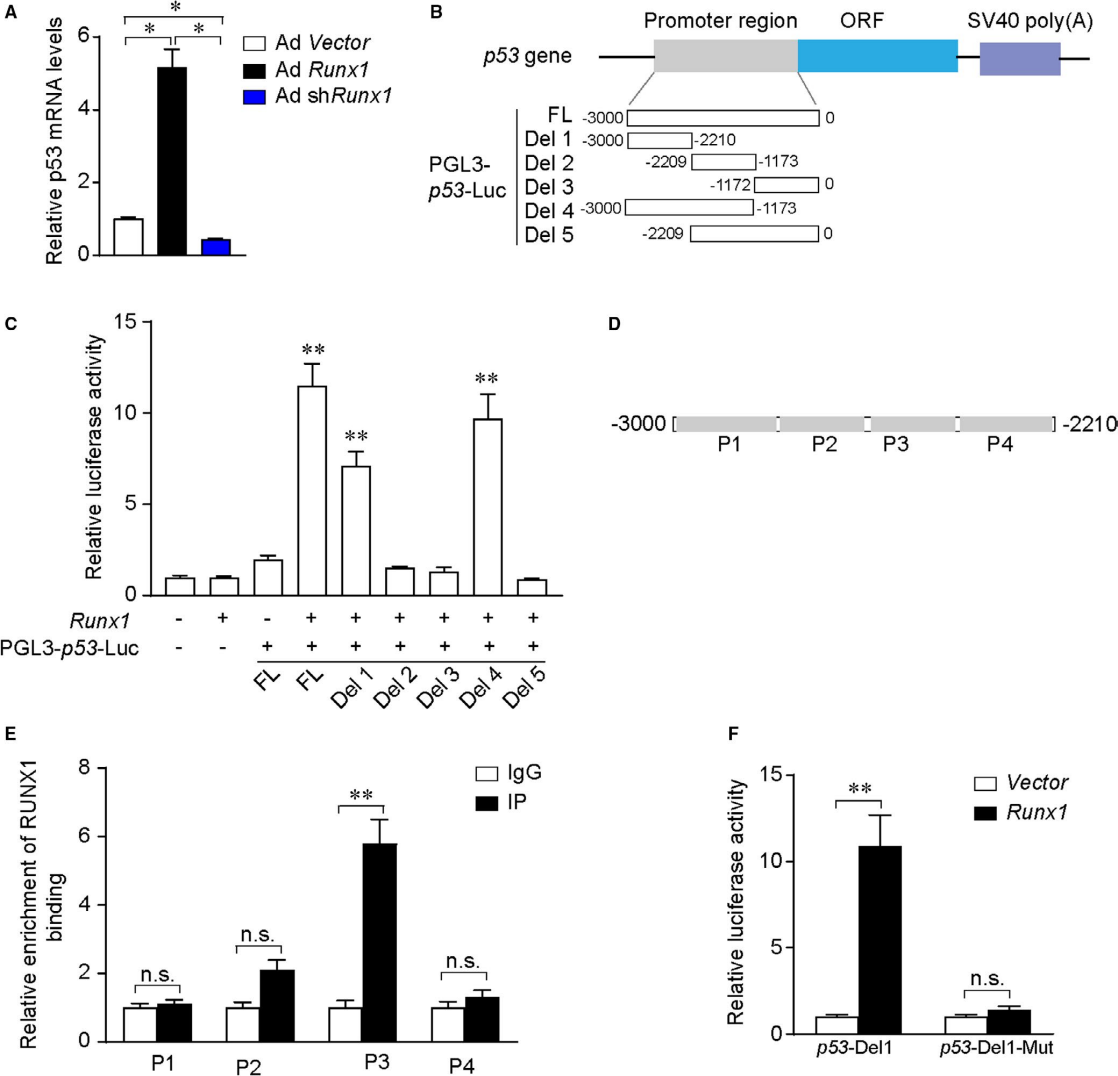
Analyses of transcriptional regulation of p53 by Runx1. (A) Representative mRNA level of Runx1 in neonatal rat cardiomyocytes (NRCMs) treatment with Ad Runx1 or Ad shRunx1 for 24 h (n = 3 samples per group). (B) Schematic images of PGL3‐p53‐Luc expression vectors containing the relevant mutations. (C) pcDNA‐Runx1 and PGL3‐p53‐Luc expression vectors containing the relevant mutations were co‐transfected into HEK293T cells. Cell lysates were collected at 24 h, and luciferase activity was measured using a luciferase reporter assay. (D) Schematic diagram of the primer locations of P1, P2, P3 and P4. (E) Quantitative PCR (qPCR) results of the chromatin immunoprecipitation (ChIP) assay results. (F) Relative values of the corresponding luciferase activities. *P < .05, **P < .01

To confirm that Runx1 contributes to p53 transcriptional regulation, we used qRT‐PCR of cardiomyocytes infected with Ad Runx1 or Ad shRunx1. As shown in Figure 5A,overexpressed Runx1 increased p53 mRNA expression, while knockdown of Runx1 down‐regulated p53 expression. We next utilized luciferase assays to further explore the mechanism by which Runx1 regulates p53 expression. Since there was no consensus sequence for Runx1 binding in the promoter region of the p53 gene, we generated five types (full‐length and a series of mutants) of luciferase reporter genes covering this region (Figure 5B). According to the luciferase assays results, Runx1 significantly increased p53‐Luc activity. Moreover, the Runx1 binding sites locate in the −3000bp ~ −2210bp region of p53 gene (Figure 5C). To further verify the direct regulatory role of Runx1 in p53 expression, we introduced chromatin immunoprecipitation (ChIP) assay. Runx1 binding sites in the promoter region of p53 (−3000bp ~ −2210bp) were divided into four fragments (P1 ~ P4) (Figure 5D). Our ChIP results showed that Runx1 binding sites were enriched in the P3 region, suggesting that the P3 region contains the binding site for Runx1 (Figure 5E), since Runx1 binding motif (TGTGGTT) has been documented in the JASPAR database. By analysing the sequence of the P3 region, we found that the P3 region only contains one Runx1 binding motif. To further verify whether Runx1 directly binds to the potential binding motif in the P3 region, we cloned the wild‐type p53 promoter (p53‐Del1) or the promoter with deletion of the Runx1 binding motif into a pGL3 luciferase reporter vector ((p53‐Del1‐Mut)). As expected, the decline in p53 promoter activity in plasmid that deletion of the Runx1 binding motif demonstrated the importance of the putative binding motif in P3 region in Runx1‐mediated p53 promoter activation (Figure 5F). Collectively, these data indicated that Runx1 directly binds to the promoter region of p53 and controls its expression.

### 3.6 Runx1 promotes cardiac hypertrophy in a p53‐dependent manner

**FIGURE 6:**
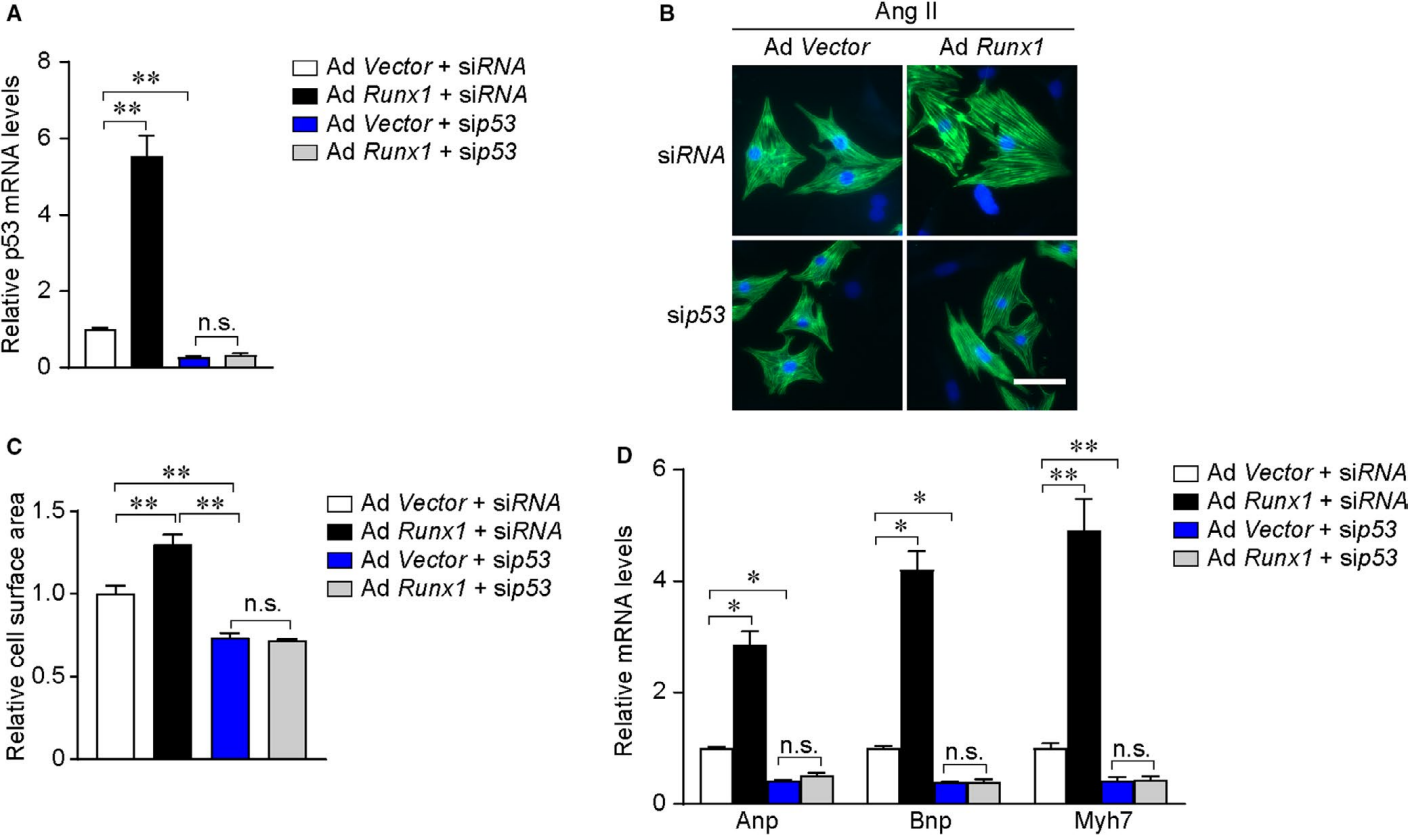
p53 is required for Runx1‐induced cardiomyocyte hypertrophy in vitro. (A) Representative mRNA level of p53 was detected after treatment with the indicated adenovirus for 24 h (n = 3 per group). (B) Representative images of neonatal rat cardiomyocytes (NRCMs) infected with the indicated adenoviruses and treated with Saline or Ang II for 48h. Cardiomyocytes were identified by a‐actinin staining (green), and nuclei were stained with DAPI (blue) (scale bars, 50 μm). (C) Quantitative results of the cell surface area of NRCMs (n ≥ 200 cells/group). (D) Representative mRNA levels of atrial natriuretic peptide (Anp), B‐type natriuretic peptide (Bnp), and Myh7 in the indicated groups. n = 4 samples per group. *P < .05, **P < .01

Because p53 seemed to be a strong candidate to mediate Runx1‐driven cardiac hypertrophy, we sought to study whether Runx1 promotes cardiac hypertrophy was p53‐dependent in vitro. We performed rescue experiments by inhibiting p53 expression through small interfering RNA method and forcing Runx1 expression in NRCMs. qRT‐PCR results showed sip53 significantly inhibited 53 expression (Figure 6A). As expected, in vitro phenotype measurements showed that inhibiting p53 expression suppressed Ang II–induced cardiomyocyte hypertrophy, which was in agreement with previous studies. Remarkably, knockdown of p53 expression mostly abrogated the ability of Runx1 to promote cardiomyocyte hypertrophy as evidenced by measuring the cell surface area and determining the mRNA levels of hypertrophic markers (Figure 6B‐D). These results indicate that Runx1 promotes cardiac hypertrophy in a p53‐dependent manner.

### 3.7 Ro5‐335, a Runx1 core binding factor β inhibitor, inhibits pathological cardiac hypertrophy

**FIGURE 7:**
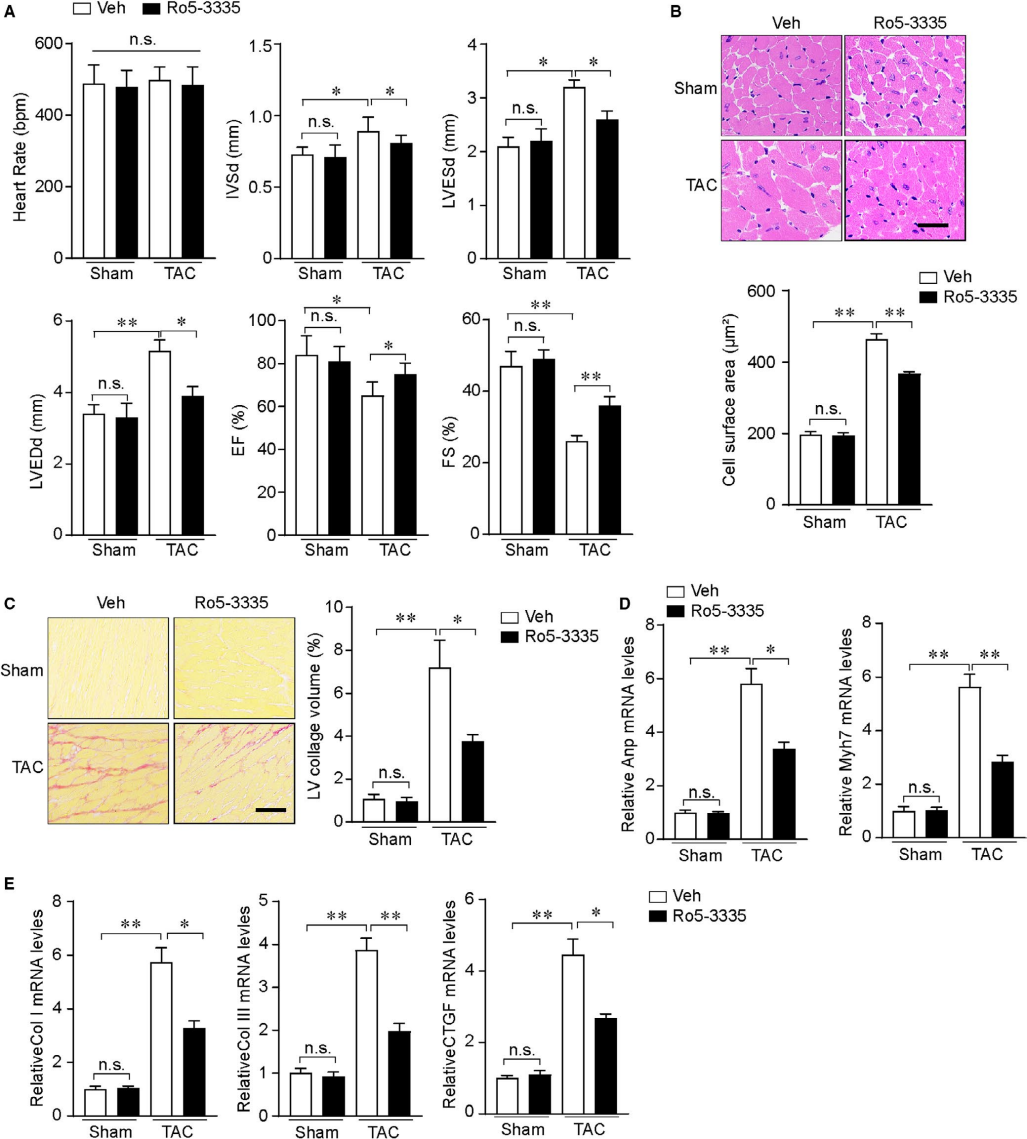
Ro5‐3335 inhibits pathological cardiac hypertrophy. (A) Statistical results of echocardiographic parameters in the indicated groups (n = 7‐10 mice per group). (B) Upper, representative images of haematoxylin and eosin (HE)–stained heart sections from the indicated groups (scale bar, 50 μm) (n = 4‐6 mice per group). Low, statistical results of cardiomyocyte cross‐sectional area in the indicated groups (n ≥ 50 fields per group). (C) Left, representative images of picrosirius red (PSR)–stained heart sections from the indicated groups (scale bar, 100 μm.) (n = 4‐6 mice per group). Right, statistical results of left ventricle collagen volume determined by picrosirius red staining. (D) The relative mRNA levels of atrial natriuretic peptide (Anp) and Myh7 in the left ventricle of mice from the indicated groups (n = 4 mice per group). (E) The relative mRNA levels of collagen I (Col I), collagen III (Col III) and connective tissue growth factor (CTGF) in the left ventricle of mice from the indicated groups (n = 4 mice per group). *P < .05, **P < .01

To investigate the potential therapeutic relevance of Runx1, the small molecule, Ro5‐3335 (a Runx1 core binding factor β inhibitor) was introduced in our in vivo experiments. Results showed that Ro5‐3335 could protect against pressure overload–induced cardiac hypertrophy as evidenced by measuring the mice phenotype, including echocardiographic measurements of LV dimensions and systolic function, histological examinations of hypertrophy, fibrosis and mRNA levels of hypertrophic and fibrosis markers (Figure 7). In summary, these results revealed that Ro5‐3335 acted as a potential therapeutic drug for treating pathological cardiac hypertrophy.

## 4 DISCUSSION

Pathological cardiac hypertrophy along with cardiomyocyte hypertrophy and fibrosis is an important pathological feature of heart failure. p53 has been shown to mediate DNA damage response and apoptosis, playing a pivotal role in inducing pathological cardiac hypertrophy.^21^ Runx1, as an important transcription factor, modulates the transcription of p53.^22^ Whether Runx1 participates in regulating pathological cardiac hypertrophy remains unclear. Our study first systematically evaluated the function and molecular mechanisms of Runx1 in cardiac hypertrophy, and we obtained some meaningful and innovative results. First, we demonstrated Runx1 as a critical positive regulator of pathological cardiac hypertrophy for the first time. Second, although the Runx1‐p53 signal pathway has been investigated in other disease model, we first discovered the binding motif of Runx1 in the promoter region of p53. Third, we first demonstrated that when use Ro5‐3335 (a Runx1 inhibitor), pathological cardiac hypertrophy could be alleviated.

Runx family is composed of three members, including Runx1, Runx2 and Runx3. Although Runx1 expression in the adult heart is reported to be low, several studies have indicated that Runx1 expression increased in the context of cardiac pathology.^14^, ^23^ Our study provides the evidence that Runx1 expression significantly increased in mouse heart tissue and cardiomyocytes in response to hypertrophic stimuli. Runx1 was originally identified a highly conserved transcription factor that modulates the haematopoiesis, inflammatory response and the immune responses.^10^, ^24^ The functional relevance of Runx1 expression in cardiac hypertrophy was unknown. In the present study, we found increased expression of Runx1 significantly promoted cardiomyocyte hypertrophy in vitro. Furthermore, knockdown of Runx1 dramatically inhibited pathological cardiomyocyte enlargement, cardiac dysfunction and fibrosis induced by chronic pressure overload. These findings indicate that Runx1 controls the progression of pathological cardiac hypertrophy and arise our great interest in exploring the underlying mechanisms. Recently, a growing body of research also implicates Runx proteins as regulators of the DNA damage response, often acting in conjunction with the p53 pathways.^9^, ^25^, ^26^ We thus hypothesis Runx1 might participate in p53‐dependent pathways in cardiac hypertrophy. Findings from our study demonstrated that Runx1 promotes pathological cardiac hypertrophy by directly binding to the promoter region of p53 and controls its expression.

Mounting evidence has demonstrated that p53 acts as a master regulator of the cardiac transcriptome and controls the progression of pathological cardiac hypertrophy.^27^ In the present study, we observed that p53 expression increased in the pathological cardiac hypertrophy and aggravated the hypertrophic responses, which was in accordance with previous studies.^28^, ^29^, ^30^ Rescue experiments further indicate that p53 is required for Runx1‐induced cardiac hypertrophy. These results indicate that the anti‐hypertrophic property of Runx1 is largely p53‐dependent. Thus, modifying the transcription activity of p53 seems like a promising strategy for treating cardiac hypertrophy.

Targeting transcription factors has become a realistic option with increased understanding of transcription factor biology and technological advances.^31^, ^32^ Since Runx1 has a crucial function in promoting cardiac hypertrophy, novel therapies targeting Runx1 may achieve an efficacious therapeutic response. Importantly, the small molecule, Ro5‐3335 was identified as a disruptor of the CBFβ–Runx1 interaction and was shown to repress CBFβ/Runx1‐dependent transactivation and inhibit a variety of Runx1‐dependent biological processes by altering the conformation of the CBFβ–Runx1 complex.^18^, ^33^, ^34^, ^35^ However, direct role of Ro5‐3335 in pathological cardiac hypertrophy has not been well documented; thus, whether Ro5‐3335 has crucial effects on cardiac hypertrophy is worth pursuing. Our experiments demonstrated that Ro3‐3335 dramatically reduced chronic pressure overload–induced cardiac hypertrophy, which suggests Ro5‐3335 acted as a potential therapeutic drug for treating pathological cardiac hypertrophy.

In conclusion, we provide evidence that Runx1 expression increased in pathological cardiac hypertrophy. Findings from this study demonstrate Runx1 as a critical positive regulator of pathological cardiac hypertrophy. Runx1 functions as a transcription factor that binds the p53 gene and promotes its expression. Furthermore, we first revealed targeting inhibition of Runx1 is a promising therapeutic strategy for treating pathological cardiac hypertrophy and heart failure.

## CONFLICT OF INTEREST

The authors declare no conflict of interest.

## AUTHOR CONTRIBUTION

**Dianhong Zhang:** Data curation (equal); Resources (equal). **Cui** **Liang:** Formal analysis (equal); Writing‐original draft (equal). **Pengcheng Li:** Data curation (equal); Software (equal). **Xiaoxu Tian:** Methodology (equal); Visualization (equal). **Zhengyang Hao:** Data curation (equal); Resources (equal). **Chenran Guo:** Methodology (equal); Software (equal). **Lingyao Kong:** Formal analysis (equal); Writing‐review & editing (equal). **Lulu Yang:** Project administration (equal); Visualization (equal). **Jianzeng Dong:** Conceptualization (equal); Writing‐review & editing (equal). **Yanzhou Zhang:** Investigation (equal); Visualization (equal). **Binbin Du:** Project administration (equal); Supervision (lead).

## Supporting information

Fig S1

Fig S2

Fig S3

Table S1

## Data Availability

The data will be made available after been required upon request from the corresponding author.
