# Supplementary figures and images for: Runt‐related transcription factor 1 (Runx1) aggravates pathological cardiac hypertrophy by promoting p53 expression
Source: J Cell Mol Med. 2021 Jun 30;25(16):7867–77. doi: 10.1111/jcmm.16704 (PMC8358850; doi:10.1111/jcmm.16704)

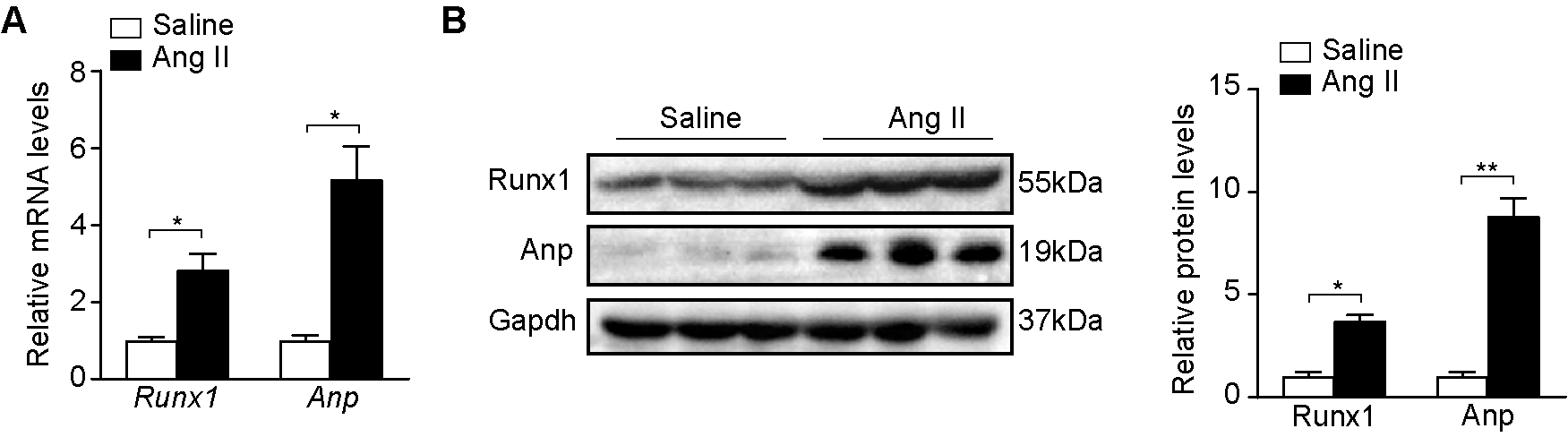

Supplement: Supplementary file 1 — Fig S1 [file JCMM-25-7867-s001.tif]

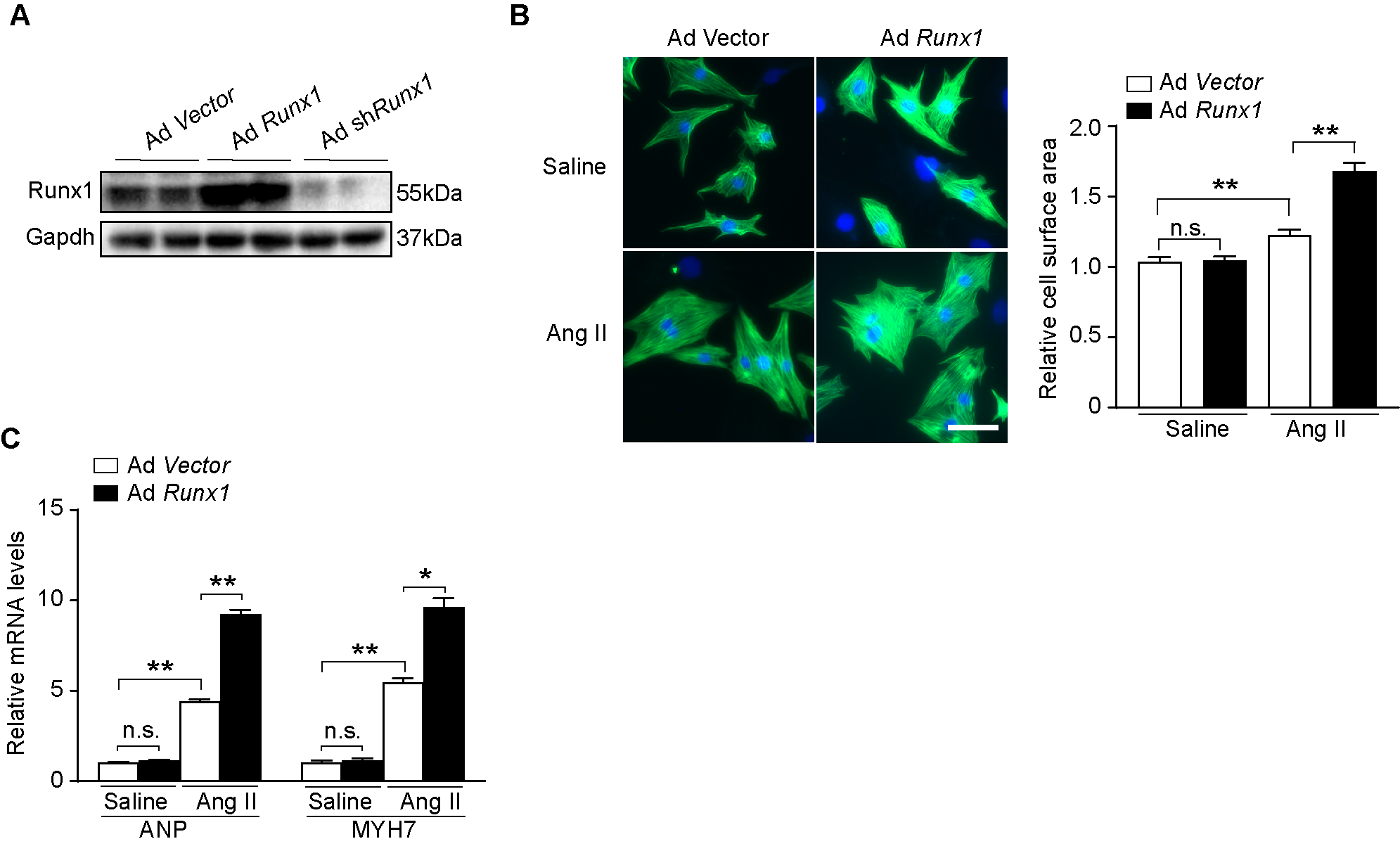

Supplement: Supplementary file 2 — Fig S2 [file JCMM-25-7867-s002.tif]

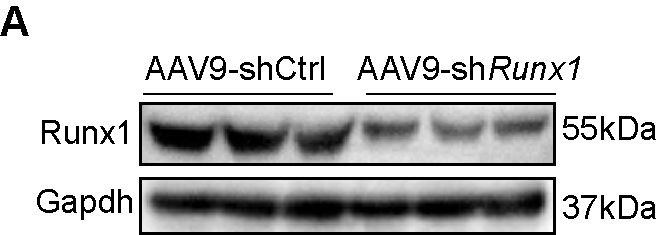

Supplement: Supplementary file 3 — Fig S3 [file JCMM-25-7867-s003.tif]
