# Supplementary material for: Runt‐related transcription factor 1 (Runx1) aggravates pathological cardiac hypertrophy by promoting p53 expression
Source: J Cell Mol Med. 2021 Jun 30;25(16):7867–77. doi: 10.1111/jcmm.16704 (PMC8358850; doi:10.1111/jcmm.16704)
Supplement: Supplementary file 4 — Table S1 [file JCMM-25-7867-s004.docx]

**Table S1. Primers used used for RT-PCR.**

|  | Forward | Reverse |
| --- | --- | --- |
| Mouse Runx1 | TGGTGGAGGTACTAGCTGACC | CGAGTAGTTTTCATCGTTGCCTG |
| Mouse Anp | GCTTCCAGGCCATATTGGAG | GGGGGCATGACCTCATCTT |
| Mouse Bnp | GAGGTCACTCCTATCCTCTGG | GCCATTTCCTCCGACTTTTCTC |
| Mouse Myh7 | ACTGTCAACACTAAGAGGGTCA | TTGGATGATTTGATCTTCCAGGG |
| Mouse Col I | AGGCTTCAGTGGTTTGGATG | CACCAACAGCACCATCGTTA |
| Mouse Col III | AAGGCTGCAAGATGGATGCT | GTGCTTACGTGGGACAGTCA |
| Mouse Ctgf | AGGGCCTCTTCTGCGATTTC | CTTTGGAAGGACTCACCGCT |
| Mouse Gapdh | ACTCCACTCACGGCAAATTC | TCTCCATGGTGGTGAAGACA |
| Rat Runx1 | ACTACCCAGGCGCCTTCACCT | CCCACCATGGAGAATTGGTAGGA |
| Rat Anp | AAAGCAAACTGAGGGCTCTGCTCG | TTCGGTACCGGAAGCTGTTGCA |
| Rat Bnp | CAGCAGCTTCTGCATCGTGGAT | TTCCTTAATCTGTCGCCGCTGG |
| Rat Myh7 | TCTGGACAGCTCCCCATTCT | CAAGGCTAACCTGGAGAAGATG |
| Rat p53 | CAACGTCTTATCCGGGTGGAAG | TGGAGTCTTCCAGCGTGATGA |
| Rat P1 | CCAGCCTCAAGCTTCCAGAGA | GTGTGCAAATGCATGTCAGTAAG |
| Rat P2 | ACCCAGAAAAACACAACCAGC | AGAGGTCATGAGTTCAATTCCC |
| Rat P3 | AGAGCAGTTGGGTGCTCTT | CTTACTGGTAATCACAGCTCTCC |
| Rat P4 | AGCACACCCAGCTGATGCA | TGAAAGACTAGTCAGTCTTTCTG |
| Rat Gapdh | GACATGCCGCCTGGAGAAAC | AGCCCAGGATGCCCTTTAGT |
